# From Abstract to Domain-Specific: Development and Validation of Matrix Reasoning Tasks for Students in Biology

**DOI:** 10.3390/jintelligence14040069

**Published:** 2026-04-17

**Authors:** Colin Peperkorn, Claas Wegner

**Affiliations:** Faculty of Biology, Biology Didactics-Giftedness and Talent Research, Bielefeld University, 33615 Bielefeld, Germany; claas.wegner@uni-bielefeld.de

**Keywords:** matrix reasoning test, giftedness, biology, STEM, test development, IRT

## Abstract

Matrix reasoning tests are frequently used to measure intelligence and identify gifted students across domains. To date, there is limited evidence on the usefulness of contextualised tasks for identifying domain-specific giftedness. In the current study, matrix reasoning tasks tailored to biological contexts were developed and validated for students in grades 3–6. The tasks were evaluated across two research cycles, involving a total of *N* = 895 students (*n*_1_ = 470; *n*_2_ = 425). An item analysis based on item response theory indicated acceptable item parameters and fit indices for the final item pool. Correlation analyses revealed moderate-to-strong associations with IQ, assessed via abstract matrix reasoning, as well as with domain-specific achievement in biological inquiry processes. A known-groups comparison revealed that students identified as gifted in biology outperformed a comparison group of peers, providing preliminary known-groups validity evidence for the developed tasks. Overall, the matrix reasoning tasks tailored to biology showed acceptable psychometric properties, demonstrated positive correlations with achievement in biological inquiry, and the study provided initial evidence of their usefulness for identifying gifted students in biology.

## 1. Introduction

Intelligence (defined as Spearman’s *g*) is a main predictor of academic performance across domains (e.g., Deary et al., 2007; Watkins et al., 2007). It is considered one of the most thoroughly researched psychological constructs and thus offers a strong foundation for identifying gifted students (Rost & Sparfeldt, 2017). Although a modern understanding of giftedness goes beyond above-average intelligence, many definitions see it as a necessary condition or potential that enables individuals to contribute to societal development through their actions (e.g., Subotnik et al., 2011; Worrell et al., 2019). Furthermore, it is essential to consider domain-specific abilities, psychosocial skills, motivation, and environmental influences, along with strategies to address these factors, as they enable the individual to translate potential into transformative giftedness (Sternberg, 2024). Subotnik et al. (2011) provided the following definition:

Giftedness is the manifestation of performance or production that is clearly at the upper end of the distribution in a talent domain even relative to that of other high-functioning individuals in that domain. Further, giftedness can be viewed as developmental, in that in the beginning stages, potential is the key variable; in later stages, achievement is the measure of giftedness; and in fully developed talents, eminence is the basis on which this label is granted. Psychosocial variables play an essential role in the manifestation of giftedness at every developmental stage. Both cognitive and psychosocial variables are malleable and need to be deliberately cultivated.

(p. 7)

Therefore, identification processes must consider both potential and achievement, must be designed in accordance with educational goals, and employ multiple stages to shape programmes that foster learning strategies, motivation, and interest in specific domains (VanTassel-Baska, 2005, 2021). On the potential stage, broad cognitive abilities, such as fluid reasoning, should be measured to identify gifted individuals. However, current instruments mainly consist of domain-general tasks or abstract forms, which do not fully align with the domain-specific nature of giftedness. In the current study, contextualised matrix reasoning tasks for biology were developed and tested with students in grades 3–6 to assess their psychometric properties and ability to identify the potential of gifted students in biology.

Intelligence should be viewed not merely as a psychological attribute but as a network of diverse individual strengths and weaknesses across different broad cognitive abilities (McGrew et al., 2023). Fluid intelligence (*G_f_*) is the reasoning ability that enables individuals to handle unfamiliar situations or problems and refers to the ability to solve problems and to recognise patterns or relations (Cattell, 1963; Schneider & McGrew, 2022). It is usually measured using matrix reasoning tests. These tests involve tasks in which a matrix, typically 3 × 3 or 2 × 2, or a row of fields (e.g., 1 × 5) is presented, with one field left empty for participants to fill from a set of options. The completion of these tasks requires inductive, deductive, and relational reasoning, along with spatial visualisation and working memory (Carpenter et al., 1990; Prabhakaran et al., 1997). The most well-known examples are the Raven’s Progressive Matrices (RPM; Raven et al., 1998), the Culture Fair Test (CFT 20-R; Weiß, 2006), the Bochumer Matrizentest (BOMAT; Hossiep & Hasella, 2010), or the Wechsler scales (WISC-V, WAIS-5; Wechsler, 2014, 2024).

Research at a domain-specific level has shown that *G_f_*, assessed by matrix reasoning tests, plays a significant role in both mathematical and verbal skills (Peng et al., 2019). Ren et al. (2015) found that measuring fluid intelligence can provide insights into learning abilities, making matrix reasoning tests a valuable addition to educational assessments. Based on the results, educational practitioners can draw direct conclusions about important aspects of their students’ learning (Klauer & Phye, 2008). In addition, there is an opportunity to gain deeper insights into the potential of their students beyond measures of academic achievement or performance (Freund & Holling, 2011a, 2011b). Given this strong empirical foundation and the large number of available tests, educators rely heavily on general intelligence tests to identify gifted students. Nevertheless, researchers emphasise the importance of considering domain-specific skills, creativity, and non-intellectual factors such as special interests and motivation when identifying gifted students (Subotnik et al., 2011). The main goal of gifted education is to foster all students according to their individual potential, considering real later-life outcomes (Sternberg, 2002). This understanding aligns with the non-*g* psychometric network analysis (PNA) approach in contemporary intelligence research, which posits that intelligence is formed through the interaction of multiple broad abilities (e.g., McGrew et al., 2023). Therefore, identifying giftedness should not rely on measuring a single, unidimensional construct to differentiate between gifted and non-gifted individuals. This also means that gifted students do not have to perform above average on every metric used (as a high IQ would require; Peters et al., 2020). Instead, the individual expression of giftedness should be identified and fostered. To achieve this, broad cognitive abilities, such as *G_f_*, must be assessed inclusively, culturally sensitive, and fairly across all students (Holden & Tanenbaum, 2023).

Gifted individuals interested in a specific area are more likely to use their potential to develop domain-specific skills. Therefore, these are essential for giftedness identification because they indicate the development and expression of potential in a specific area of interest (Subotnik et al., 2011; VanTassel-Baska, 2005). The ability to perform scientific inquiry processes, epistemological views (Nature of Science), and practical skills (scientific working techniques) are core competencies in biology (Arnold et al., 2014; Wellnitz & Mayer, 2011). These include scientific reasoning skills like formulating questions and hypotheses, planning experiments, and drawing conclusions, as well as inquiry methods such as skills in observing, collecting data, or systematically controlling and varying variables in experiments or models (e.g., Bell et al., 2010; Bybee et al., 2009; OECD, 2023; Pedaste et al., 2015). Research and debates on direct relationships between (fluid) intelligence and domain-specific abilities also extend to STEM subjects (science, technology, engineering, and mathematics; Brookman-Byrne et al., 2019; Richland et al., 2007; Yuan et al., 2006). Greiff and Neubert (2014), in a study with 490 high school students, found evidence for the relationships between fluid intelligence, measured with the CFT 20-R (Weiß, 2006), and complex problem solving (CPS) in scientific inquiry processes, as a domain-specific ability, assessed using MicroDYN tasks (Greiff et al., 2012). Scherer and Tiemann (2014) confirmed the relationship between fluid intelligence and CPS in science among students in grades 8 to 10. Other studies suggested that intelligence has only weak to moderate correlations with ability domains such as scientific reasoning (Sternberg et al., 2019). It is noted that the strength of the correlation is highly dependent on the measurement method. The results indicate that, for matrix reasoning tests to be used meaningfully in domain-specific research and identification processes, they must be valid and reliable (Van Hoogdalem & Bosman, 2024).

At the potential level, mainly domain-general measures are currently used in giftedness identification processes, which are mostly supplemented with domain-specific achievement measures to serve the educational purpose. One emerging option is to contextualize intelligence measures within a specific field, thereby integrating domain-general and domain-specific cognitive processes (Roberts, 2007). In their study, Benit and Soellner (2012) adapted a matrix reasoning test for mechanical engineering and, with a sample of 360 university students, demonstrated that participants’ willingness to complete the test could be significantly increased. The measurement of domain-specific cognitive processes at the potential level currently receives little attention in giftedness identification and there is a lack of validated, school-age, domain-specific matrix reasoning tests that accurately measure fluid reasoning.

In the current study, a domain-specific matrix reasoning test in biology was developed within a design-based research (DBR) framework to improve identification processes of gifted students in schools (Peperkorn & Wegner, 2024). The DBR framework involves identifying a problem in educational practice, developing a prototype to address it based on a preliminary examination, and then evaluating and refining it through multiple research cycles. This process yields both practical outputs and contributes to existing theories (Euler, 2014; Shavelson et al., 2003). Therefore, matrix reasoning tasks were designed with biological themes to assess students’ fluid reasoning abilities in grades 3–6 using a domain-specific approach. The age group was selected because, during the transition from primary to secondary school, a decline in interest in science is observed (Gebhard et al., 2017; Potvin & Hasni, 2014). In Germany, students typically transition between 4th and 5th grade, although in two states, it occurs after sixth grade. Identifying and fostering gifted students is particularly important during this phase. The assessment is intended for classroom group administration in low-stakes environments. The primary objective of the study was to pilot the test, investigate its psychometric properties, and assess the quality of the developed items. Different forms of the newly developed matrix test were used in two studies. The matrix reasoning tasks was administered across different cohorts, and accompanying instruments were used to gather preliminary evidence of validity. References to IQ and skills in scientific inquiry processes in biology were examined to analyse subject-specific contextualisation and the associations with subject-specific achievement. In addition, group comparisons were conducted to examine the suitability for subject-specific giftedness identification. The following research questions were posed:

**RQ1.**  *What is the psychometric quality of the developed domain-specific matrix reasoning tasks across both test versions?*

**RQ2.**  *How are the results of the domain-specific matrix reasoning tasks related to IQ and abilities in scientific inquiry processes in biology?*

**RQ3.**  *Do gifted students in an enrichment program show different ability levels in completing domain-specific matrix reasoning tasks compared to a control group?*

## 2. Materials and Methods

The present study is a quantitative cross-sectional study aimed at examining the psychometric quality of domain-specific matrix reasoning tasks. The study was conducted in Germany as part of an enrichment program to foster gifted students in biology (Wegner et al., 2013) and in cooperating schools of the project. Two research cycles were conducted following the DBR methodology (Peperkorn & Wegner, 2024; Euler, 2014; Shavelson et al., 2003). In the first study, a 24-item test version was administered. In the second study, an expanded version comprising 60 items was used.

### 2.1. Participants

The total sample consisted of *N* = 895 students (41.7% female, 54.8% male, 3.5% N/A, mean age = 10.1 years) from the third to sixth grade. The overall sample was divided into two studies. After piloting the initial version, a second expanded test version was used. In both samples, the participants stemmed from two separate cohorts. The first cohort consisted of participants in an enrichment program (Wegner et al., 2013) who were identified by their biology teachers. The second cohort was assembled from students attending partner schools. We used a non-probability convenience sample recruited through collaborating teachers. Students were recruited from primary schools (grades 1–4) and from secondary schools, namely academic-track secondary schools (Gymnasium; grades 5–13), from urban and rural areas. No participants of the enrichment program were part of the second cohort.

The sample of the first study included *n*_1_ = 470 students (42.3% female, 55.7% male, 2.0% N/A, mean age = 10.08 years). Of these, 373 were participants from the enrichment program, and 97 were students from participating schools. In the first study, we contacted six teachers from three different schools within our collaboration network. Three teachers agreed to participate, yielding a 50% participation rate. This resulted in four classes (grades 5–6) participating. All students present in the participating classes were invited. A total of 104 students were eligible, 99 provided parental consent, and 97 completed the assessment, resulting in a student participation rate of 93.3%. Two students who provided consent were absent during the study.

The sample of the second study included *n*_2_ = 425 students (40.9% female, 53.9% male, 5.2% N/A, mean age = 10.12 years). Of these, 341 were participants from the enrichment program, and 84 were students from participating schools. In the second study, we contacted three teachers from two different schools. All teachers agreed to participate, yielding a 100% participation rate. This resulted in three classes (grades 5–6) in which all students present were invited. A total of 87 students were eligible, 84 provided parental consent, and all completed the assessment, resulting in a student participation rate of 96.6%.

### 2.2. Data Collection Tools

#### 2.2.1. Domain-Specific Matrix Reasoning Test for Biology

**Figure 1:**
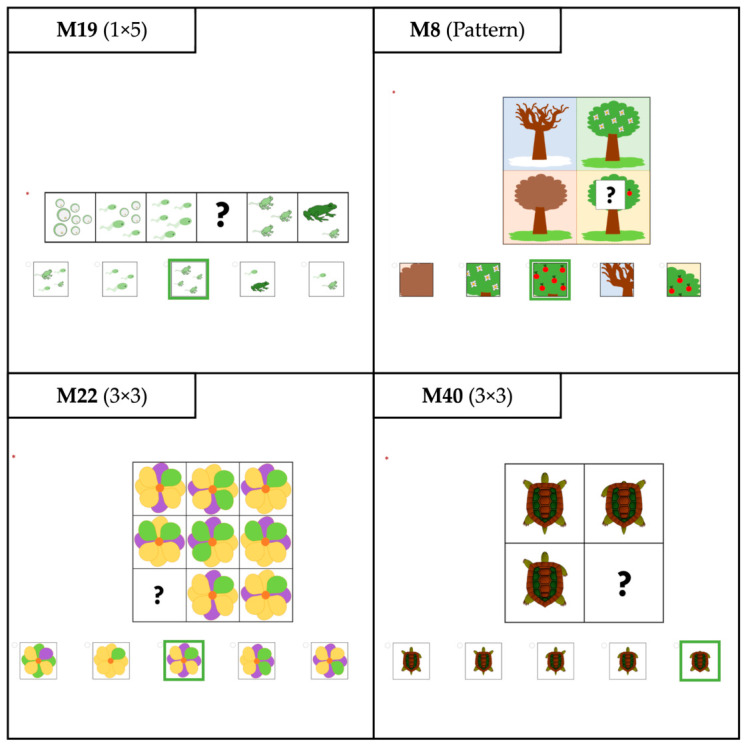
Examples of the developed domain-specific matrix reasoning tasks. Correct answers are outlined in green.

Domain-specific matrix reasoning tasks with biological references were developed. Biological forms, such as animals, plants, natural phenomena, or laboratory materials, replaced common abstract shapes. Items were created in four formats (see Figure 1), including 2 × 2, 3 × 3, and 1 × 5 matrices, as well as patterns with cut-out fields to be completed (Matzen et al., 2010). Five answer options were created for each item, including one correct answer and four distractors. This choice aligns with standard practices in school-age matrix reasoning tests (e.g., WISC-V; CFT 20-R; RPM). To ensure fairness across diverse student groups, the nonverbal items were designed so that their solutions do not require prior knowledge. The items can be enlarged at will or displayed in grayscale. For the first version, 24 items were developed. These were presented in a fixed order in the first study. In the second study, the initial item pool of 24 items was expanded to 60 items, and the items were presented in randomised order. The students were introduced to the items by answering trial items of each type and receiving automatic feedback on whether their answers were correct. Item 1 was used as an example during test administration and was therefore excluded from all analyses. The items were created and administered digitally using LimeSurvey, with processing on tablets (2732 × 2048 pixels). During administration, participants received no feedback on the correctness of their answers and were unable to skip items or revise submitted answers. Participants were given 20 min to complete the test in both versions.

#### 2.2.2. Raven’s Progressive Matrices 2, Clinical Edition—German Short Form (NCS Pearson, 2019)

Participants’ IQ was assessed using the German digital short form of Raven’s Progressive Matrices (NCS Pearson, 2019). The matrix reasoning test is used to evaluate overall cognitive abilities, with a primary focus on fluid intelligence. It has been validated with a European norm sample and is appropriate for assessing individuals aged between 4:0 and 69:11 years. The digital short form demonstrated a test-retest reliability between *r* = 0.79 and *r* = 0.81 (NCS Pearson, 2019). The test duration is limited to 20 min.

#### 2.2.3. Abilities in Scientific Inquiry Processes in Biological Research Contexts

The assessment was used to evaluate the student’s abilities in scientific inquiry processes as domain-specific abilities. Scientific inquiry was assessed using the VerE model (Nowak et al., 2013). This theoretical framework encompasses scientific reasoning, including the ability to formulate hypotheses and research questions, plan and perform investigations, and draw conclusions; as well as inquiry methods such as observing, comparing, and arranging, experimenting, and modelling, as overlapping dimensions. The items consisted of a brief description, a visualisation, and a question about the biological phenomenon, model, or experiment. The instrument comprised 54 multiple-choice items, of which 18 were administered in a digital test version. The test instrument was developed as part of the research project. Item analyses were conducted and psychometric properties were assessed for the current sample (grades 3–6; KR-20 = 0.632–0.742). DIF analyses for gender and grade level, divided by student group, showed no significant DIF for any of the items. A translated example was as follows: “A chameleon is observed in its terrarium. You can see that the chameleon turns darker as soon as a conspecific approaches, it is fed, or touched. Which assumption can be verified through the described observation? The chameleon (a) changes its colour in different situations to communicate (correct answer); (b) only changes colour when threatened; (c) only changes colour when it is hungry; (d) becomes brighter when it is touched.” To prevent excessive demands through the description texts, a read-aloud function has been implemented. The assessment was also administered via LimeSurvey and took approximately 25 min to complete.

### 2.3. Procedure

The administration was conducted in two different settings. The cohort of participants in the enrichment program was surveyed during their project courses, with groups consisting of 15–20 students. The participants were equipped with tablets. The control cohort completed surveys in groups of 25–30 students during lessons at the participating schools. If school-owned tablets were available, they were used. Otherwise, the research team provided participants with tablets. The surveys were conducted following a standardised procedure. Each survey session took approximately 30 min, including greetings, introductions, process explanations with a trial item, execution, and farewell. Testing was conducted by trained research staff using a standardised protocol. To ensure comprehension, examiners read the general instructions aloud to all students, administered trial items with feedback, and explained that students could choose and, if necessary, change an answer before submitting. It was explained that submitted answers cannot be corrected. During the test, procedural support was limited to rereading or paraphrasing the general instructions from the protocol, clarifying the response format (e.g., “choose the answer that completes the pattern”), and reminding students that no subject-matter knowledge was required. Examiners were explicitly instructed not to provide hints to the correct answer and not to confirm whether an answer was correct. Assistance procedures were identical across groups to preserve comparability. Written consent has been obtained from a parent or legal guardian of all participants. All participants were briefed on the purpose of the research and were informed that their participation was voluntary. They could withdraw at any time. The study was reviewed and approved by the ethics committee at Bielefeld University (approval number: 2025-256; approval date: 27 August 2025).

### 2.4. Data Analysis

To address the first research question, an item analysis using item response theory (IRT) was conducted for both test versions. A Rasch model (1PL) was estimated to determine the reliability of expected a posteriori (EAP) and mean weighted likelihood estimation (WLE; Warm, 1989), item difficulty (*b*), and the item-fit values weighted/unweighted mean-square (MNSQ) and z-standardised statistic (ZSTD). Additionally, the Kruder-Richardson 20 formula (KR-20) was used. The unidimensionality of each of the two versions was evaluated using Principal Component Analysis (PCA) of the residuals (Linacre, 1998), and local dependencies were assessed using Yen’s Q3 method (Yen, 1984). In both versions, the PCA showed that the eigenvalue of the first residual contrast was <2.0, indicating that the residuals do not form a meaningful secondary dimension and thus support unidimensionality. For the initial version, Yen’s Q3 method showed a raw mean Q3 of −0.04 and an adjusted mean Q3 of less than 0.001. Item-specific analysis showed that all mean absolute Q3 values were <0.20 (Max = 0.112) with a maximum of two violations observed across all item pairs. Similarly, for the second version, Yen’s Q3 method showed a raw mean Q3 of −0.018 and an adjusted mean Q3 of less than 0.001. Item-specific analysis showed that all mean absolute Q3 values were <0.20 (Max = 0.077), with a maximum of two violations observed across all item pairs as well. Given these generally low residual correlations, the assumption of local independence was considered to be sufficiently met in both versions, and unidimensionality was further verified. In addition, uniform differential item functioning (DIF) analyses for the second version of the matrix reasoning test were conducted across the different cohorts (enrichment/control), genders (male/female), and school levels (primary/secondary level). We have decided to use school levels rather than grade levels to align the analysis with the key curricular transition and to better capture children’s progress over time. Grade-by-grade DIF may misattribute expected growth to bias and is influenced by small cell sizes in our data, thereby reducing stability and statistical power (Penfield, 2001). For this, the Mantel-Haenszel DIF procedure (MH; Mantel & Haenszel, 1959) with iterative anchor purification was used. The robustness of the results was examined using Lord’s chi-square DIF test (Lord, 1980) and Raju’s area-based DIF test (Raju, 1988). The *p*-values were adjusted using the Benjamini-Hochberg method (*α* = 0.05; Benjamini & Hochberg, 1995). DIF effect size was evaluated according to Zwick et al. (1999): Δ_MH_ units < 1 indicated a negligible effect, 1 < Δ_MH_ units < 1.5 indicated a slight to moderate effect, and Δ_MH_ units > 1.5 indicated a large effect.

For the second research question, the results of the matrix reasoning test, the scientific inquiry assessment, and the IQ were correlated, using Spearman’s *ρ* (Shapiro-Wilk: *p* < .05). For this, the person’s ability parameters (*θ*) were used for the matrix reasoning test and the scientific inquiry assessment. Not-reached responses were recorded as missing and did not affect the likelihood. The correlation analysis was conducted separately for the third and fifth grades to account for age-standardisation of the IQ data.

To answer the third research question, we estimated an exploratory latent regression Rasch model for the two different cohorts (enrichment/control). For further verification of the results, we estimated a multiple-group Rasch model using marginal maximum likelihood with EAP ability estimates. To rule out potential measurement invariance, we employed Stocking-Lord linking (Stocking & Lord, 1983), assuming complete non-invariance as a robust approach (Baghaei & Robitzsch, 2025; Robitzsch & Lüdtke, 2020). This method enables item response modelling with multiple groups across different item sets because the item parameters for both sets are brought onto a common scale. In our case, this allows us to compare both groups (enrichment/control) across both test versions.

All analyses were performed using R software (Version 4.5.2; R Core Team, 2025) and ACER ConQuest software (Version 5.47.5; Adams et al., 2020).

## 3. Results

### 3.1. Descriptive Results

Table 1 summarises the descriptive results of both test versions separated by cohorts.

### 3.2. Item Analyses

An item analysis was conducted for both versions of the matrix reasoning test to answer the first research question (see Table 2 and Table 3).

**Figure 2:**
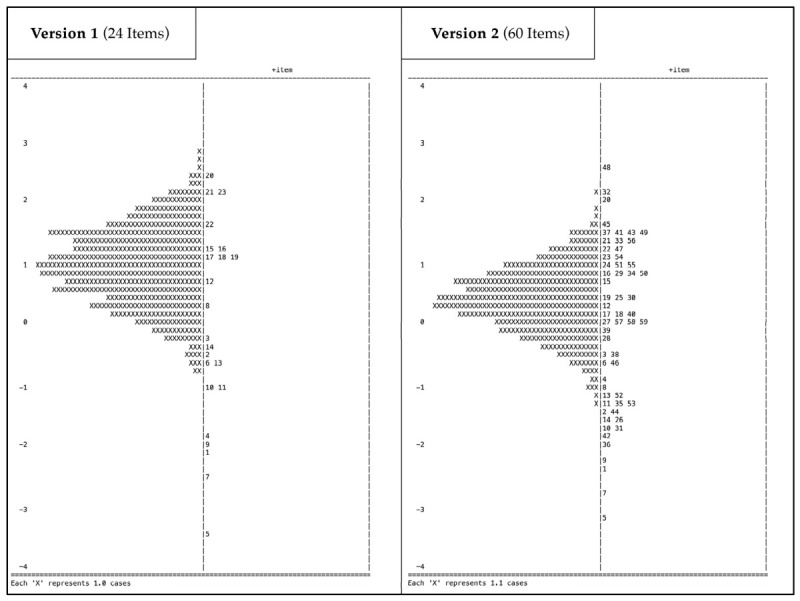
Wright maps of both evaluated matrix reasoning test versions.

The *b* values for the items in the first version (24 items) ranged from −3.39 to 2.45 (see Figure 2). All items met the MNSQ criteria for infit and outfit (0.8 ≤ MSNQ ≤ 1.2; Wright & Linacre, 1994). Item 23 showed high ZSTD values for infit and outfit, and Item 22 showed high ZSTD values for outfit (ZSTD ≤ 1.96), indicating underfit. The reliability of the first version was as follows: EAP = 0.60; WLE = 0.58; KR-20 = 0.60. In the second version, the *b* values ranged between −3.07 and 2.58 (see Figure 2). In the second version, the difficulty range was more effectively covered. Coverage was less dense toward the extremes, with only a few items in the ability ranges of *θ* < −2.0 and *θ* > 2.0. Additionally, there were fewer items in the range −1.0 < *θ* < 0. All items met the criteria for MNSQ and ZSTD values for infit. Only item 49 failed the outfit MNSQ criterion, with an unweighted MNSQ = 1.67, indicating underfit. Six items (6; 11; 12; 36; 43; 54) showed a ZSTD value below −1.96 for outfit, indicating overfit. Two items (38; 49) showed ZSTD values for outfit above 1.96, indicating underfit. The reliability of the second version was as follows: EAP = 0.74; WLE = 0.73; KR-20 = 0.75. A uniform DIF analysis was conducted for both versions of the matrix reasoning test across the two cohorts, genders, and grade levels. In the first version, notable DIF was observed for items 3 and 20, with cohort as the grouping variable. However, only item 3 also exhibited DIF in the robustness checks via Lord’s chi-square DIF test and Raju’s area-based DIF test. Both items showed a Δ_MH_ value above 1.5, indicating DIF favoring the control group of students. No significant DIF items were identified across gender or grade levels. We decided to retain item 3 in the item pool to collect additional information on potential biases. In the second version 2, no significant DIF items were identified across all analysed groups using the MH DIF procedure (see Table 4). Regarding the DIF analysis across the two cohorts, items 12 and 15 showed Δ_MH_ values below −1.5, indicating DIF favouring the enrichment students. Items 3 and 27 showed Δ_MH_ values above 1.5, indicating DIF favouring the control group of students. All of these values were non-significant. Items 3, 12, and 33 showed significant DIF in Lord’s chi-square DIF test and Raju’s area-based DIF test, but not in the primary analysis using MH procedure. All items were included in subsequent analyses.

### 3.3. Correlational Analyses

Correlation analyses between the results of the developed matrix reasoning test (*θ*_MR_), the results for abilities in scientific inquiry processes (*θ*_SI_), and IQ were conducted separately for primary and secondary grade levels (see Table 5). The results of the primary grade level showed significantly positive moderate correlations (*ρ* > 0.3) between *θ*_MR_ and IQ, and between *θ*_MR_ and *θ*_SI_. A significantly positive weak correlation (*ρ* > 0.1) was observed between *θ*_SI_ and IQ. The results for secondary-grade level showed strong, significant positive correlations (*ρ* > 0.5) between *θ*_MR_ and IQ and between *θ*_MR_ and *θ*_SI_. A significantly moderate correlation was observed between *θ*_SI_ and IQ.

### 3.4. Group Comparisons

The Rasch latent regression model included the categorical covariate cohort (enrichment vs. control; enrichment as the reference group). The estimated contrast was *β* = −0.33, indicating lower mean ability in the control group (*R*^2^_θ_ = 0.03; see Table 6).

A multi-group Rasch model with equal item parameters across groups corroborated these results. With the enrichment-group mean fixed to 0, the control group mean was −0.31 logits (*d* = −0.53; see Table 7).

## 4. Discussion

The present study investigated the psychometric properties of contextualised matrix reasoning tasks, featuring biological forms and representations, to assess students’ fluid reasoning abilities in grades 3–6 as part of giftedness identification in biology. In the following, we first discuss the findings from the correlation analysis and the group comparison, before detailing the psychometric properties of the tasks.

For the primary grade level, the correlation analysis indicated a moderate association between *θ*_MR_ and IQ. The strength of the correlation indicated at least a meaningful overlap of the tested latent abilities. The developed matrix reasoning test showed a moderate convergent validity with fluid intelligence in the primary grade level. The moderate correlation between *θ*_MR_ and *θ*_SI_, and the weaker correlation between *θ*_SI_ and IQ, suggest that the domain-specific adaptation was effective and that individuals who score higher on SI also tend to score higher in the domain-specific matrix reasoning tasks. These correlations were evident at the secondary grade level. Here, strong correlations were observed between *θ*_MR_ and IQ, as well as between *θ*_MR_ and *θ*_SI_, indicating substantial overlap and convergence. Consistent with developmental psychology research, MR correlated similarly with IQ and SI at secondary grade levels. For the MR–IQ association, adolescent development in domain-general executive functions and fluid reasoning enhances reliance on cognitive processes fundamental to both measures, such as pattern abstraction, rule induction, and working memory coordination. A shift toward more analytical, rule-based strategies also strengthens the alignment with IQ (e.g., Best & Miller, 2010). In the MR–SI correlation, secondary school science teaching develops inquiry skills such as identifying patterns, evaluating evidence, and managing variables, as well as representational fluency, such as interpreting biological diagrams and graphs. Since our MR included biology-based stimuli, the lower content novelty and common analytic demands might further connect MR to SI (e.g., Zimmerman, 2007). The higher correlations observed at the secondary level may be due to older students being more experienced in dealing with biological illustrations and common representations. Nevertheless, the newly developed test showed no redundancy with Raven’s Progressive Matrices 2 (NCS Pearson, 2019). The strong correlation between *θ*_MR_ and *θ*_SI_ confirmed the primary-grade results and indicated that adapting the matrix reasoning tasks for biology was successful.

Group comparisons between students in the enrichment program and the control group, estimated using the latent regression Rasch model, indicated that students in the enrichment program outperformed control group students. Accordingly, the measurement direction of the test instrument could be confirmed. In the Rasch latent regression that included cohort (enrichment vs. control) as a binary predictor, the estimated group effect was β = −0.33 logits, corresponding to a standardised mean difference of d ≈ 0.33, which is generally considered small to moderate (Cohen, 1988). Because the groups were unbalanced (about 75% enrichment and 25% control), the maximum explanation of between-person variance by this contrast was limited. With current proportions, an effect of this size is expected to explain about 2% of the variance (Rosenthal & Rosnow, 2008). Consistent with this, the model produced R^2^_*θ*_ = 0.03. Small differences are due to sampling variability and the fact that the latent variance was estimated rather than fixed. The estimated multigroup Rasch model confirmed the initial indications of known-groups validity. Nonetheless, to improve the discriminative ability of the test instrument, items should be prepared for adaptive testing in future studies (e.g., Weiss & Vale, 1987).

The item analysis showed that the second test version exhibited acceptable item parameters and fit indices. Reliability could be significantly improved by expanding the test instrument from the first to the second version. The reliability of the second version was within an acceptable range but should be improved through further adjustments. The targeting between item difficulty (*b*) and person ability (*θ*) was improved in the second version. Although the difficulty range was generally well covered, some gaps in item coverage at certain trait levels were still apparent (Lord, 1980; see Figure 2). Extreme items might be removed or adjusted to heighten their informational value. Considering their application in giftedness identification, high discriminative power and test information are essential for the valid use of matrix reasoning tasks (Rost & Sparfeldt, 2017). In the second version item–person targeting was enhanced by expanding coverage of item difficulties throughout the observed ability range (see Figure 2). However, coverage was less dense at the extremes and sparse in the lower mid-range. In practice, this entails a risk of reaching ceiling performance within the highest-ability subgroup and diminished discrimination for individuals with lower-to-mid abilities. To address these gaps, future revisions should expand the item pool to include very difficult items (*b* > 2.5), very easy items (*b* < −2), and a small set targeting the lower-mid range (−1.0 < *b* < 0) to ensure discrimination across all ability levels. Because of the identified targeting gaps, correlations and group comparisons should be interpreted with caution. These gaps increase the conditional standard error of the mean (SEM) and weaken group differences and correlations (Lord, 1980). The analysis of fit indices revealed that all items met the infit criterion for high-stakes tests (Wright & Linacre, 1994). A total of six items showed standardised values outside the criterion for outfit (ZSTD < −1.96), which indicated overfitting. This could enable shortening the instrument and eliminating redundant items. However, these findings require verification through additional studies. A short instrument that does not overwhelm participants would be advantageous for identifying gifted students in schools, particularly among younger age groups. The two items that showed underfitting for the outfit (ZSTD > 1.96) should be monitored in future research, as their increased difficulty may have led participants to guess excessively. Since the infit values met the criterion and difficult items are essential for talent identification to distinguish high-ability individuals, there is no reason to revise these items. For item 3, all three methods identified significant DIF across different cohorts, favouring the control group in the first version. In the second version, no significant DIF was detected for this item. However, this item warrants special attention in future studies, as do items 12, 15, and 27, where non-significant but noteworthy Δ_MH_ values were observed (see Table 4). These differences could have methodological causes. Although the instrument was administered according to a set procedure, the cohort of enrichment students was surveyed as part of the project courses. In contrast, the control group students were surveyed during lessons at school. The survey settings revealed differences in group size and atmosphere. The items in question showed extreme b-parameters, indicating they were at the lower or upper ends of the difficulty scale. The direction of DIF varied across items and did not follow a consistent pattern, suggesting that the observed effects are more likely caused by item-level properties than by a consistent group-related bias. Analyzing the cognitive processes and solution strategies associated with these items may provide more detailed explanations and help guide targeted item revisions (e.g., Laurence & Macedo, 2023). The developed matrix reasoning tasks still showed negligible or no significant DIF between the cohorts, strengthening the test instrument’s quality and practical applicability. In addition, no indications of DIF were found for gender or grade level. The developed items demonstrated acceptable characteristics for further research.

When interpreting the results of this study, several limitations should be considered. The results of the DIF analysis were obtained using the MH procedure (Mantel & Haenszel, 1959). In this method, only uniform DIF is assessed. Therefore, Lord’s chi-square test and Raju’s area method were used for robustness checks. Here, three items showed significant values, indicating possible non-uniform DIF. These items should be monitored in future studies. Although the items include contextualised forms, their solution does not require understanding the forms’ content to avoid bias against groups with different levels of knowledge. We lack information about our participants’ ethnic-racial identity and socio-economic background. To ensure the fairness of the developed test among various ethnic and racial groups, this should be examined in future research (Holden & Tanenbaum, 2023). Furthermore, only academic track schools (Gymnasium) were included among the participating schools. To develop an instrument suitable for all school types and examine potential bias, students from diverse educational backgrounds should be included in future validation efforts. Although both rural and urban schools participated in the study, potential DIF between groups from these areas warrants further investigation. This study depended on existing partnerships, which led to unequal group sizes and a non-probability, clustered sampling method. Though we standardised the administration, residual confounding could remain, and limited precision for smaller groups cannot be entirely excluded. Future research should incorporate stratified recruitment to improve balance and enhance generalisability. For further analyses addressing the second and third research questions, we decided to retain all items in the data pool to obtain a comprehensive picture of the developed items. This procedure may have introduced distortions. Slightly different results would have been obtained through a more rigorous approach and the elimination of marked items. For the correlation analysis, it should be noted that the calculated IQ was derived from the Raven’s Progressive Matrices 2—Short Form (NCS Pearson, 2019) and did not include person-ability values. Furthermore, a large proportion of students in the correlation analysis sample came from the enrichment program, possibly affecting its results. When calculating the person’s ability parameters (*θ*) for the matrix reasoning test and the scientific inquiry assessment, we treated no-response answers as missing data, which were not included in the calculations. This approach reduced information and increased SE(*θ*), but it prevented penalising construct-irrelevant non-responses and might have biased the results of the correlation analysis. Furthermore, the reliability of the instrument used to assess abilities in scientific inquiry in the present sample was questionable. When comparing the two cohorts, it is important to note that the students in the enrichment program were chosen by their biology teachers. This selection provides a weak criterion for known-groups validity, as teachers have difficulties in identifying domain-specific gifted students (Bergold, 2014; Machts et al., 2016; Urhahne & Wijnia, 2021). Additionally, the cohorts being compared differ greatly in size, resulting in significantly more comprehensive information for estimating students’ abilities in the enrichment project. The small number of control students limits the significance of the analysis and should be increased in future studies.

The results of the present study provide initial findings that the domain-specific adaptation of matrix reasoning tasks has been successful in the biological domain. Nevertheless, the backgrounds of the effect must be examined more thoroughly in subsequent studies. Experimental contrasts (e.g., contextual versus abstract matrices) should be used to assess the effects of contextualisation on solving strategies, cognitive demands, and student engagement. Additionally, automatically measured process indicators (e.g., response time; Wise & Kong, 2005) should be examined, and speededness effects should be controlled. To determine whether the stronger correlation with the domain-specific comparison instrument (SI) is attributable to domain-specific skills, knowledge, interests, or increased test-taking motivation, additional test instruments should be used. To enhance the generalisability of the results, it would be appropriate to compare them with matrix reasoning tasks adapted for other STEM subjects or with existing findings in other domains (e.g., Benit & Soellner, 2012). To further explore the initial results regarding convergent validity, additional intelligence tests (e.g., Wechsler, 2014), measures of fluid intelligence (e.g., Weiß, 2006), and measures of domain-specific abilities (e.g., Darman et al., 2024; Greiff & Neubert, 2014) should also be utilised.

In summary, the results of the present study show that the developed item pool exhibited acceptable psychometric properties. Nevertheless, further validation should be conducted in future research to examine the test instrument’s measurement quality more thoroughly and to make additional adjustments. Particular attention should be given to the appropriateness for identifying domain-specific giftedness in school. Self-report, think-aloud, or eye-tracking studies used to explore cognitive strategies could offer deeper insights (Laurence & Macedo, 2023). Furthermore, future endeavours should examine methodological decisions, such as the inability to revise answers, the lack of response feedback, and the available time (Frey et al., 2024), to continually optimise the instrument’s design (Peperkorn & Wegner, 2024; Euler, 2014; Shavelson et al., 2003). The development of an item bank that enables adaptive testing and the creation of different test versions can further improve the test’s discriminatory ability and enhance its practical usefulness for giftedness identification procedures with regard to DBR goals (e.g., Chierchia et al., 2019; Pallentin et al., 2023). In identifying gifted students, it is important to consider multiple broad ability domains (e.g., McGrew et al., 2023), specific subject skills, creativity, and non-cognitive personality traits (e.g., Sternberg, 2024). The findings of the present study support the idea that measuring the broad ability area *G_f_* with contextualised matrix reasoning tasks can potentially improve identification processes. Future work should broaden the theoretical scope by exploring transfer to other STEM contexts and conducting cross-site studies.

## Figures and Tables

**Table 1:** Descriptive statistics of both test versions.

Version	Number of Items	Cohort	*n*	Items Solved				Items Unanswered
				Mean (SD)	Median (IQR)	Min	Max	(%)
1	24	All	470	14.97 (2.96)	15 (4)	7	22	0.19
		Enrichment	373	15.52 (2.80)	16 (4)	8	22	0.25
		Control	97	12.84 (2.56)	13 (4)	7	18	0
2	60	All	425	13.40 (3.01)	13 (5)	4	20	12.20
		Enrichment	341	13.53 (2.98)	14 (4)	4	20	13.33
		Control	84	12.83 (3.09)	13 (4)	5	19	7.61

Note. SD = standard deviation; IQR = interquartile range.

**Table 2:** Item parameters and fit indices for all items of the first version of the matrix reasoning test.

Item	*b*	SE	Unweighted Fit			Weighted Fit		
			MNSQ	CI	ZSTD	MNSQ	CI	ZSTD
2	−2.02	0.20	1.18	(0.87, 1.13)	2.70	1.02	(0.66, 1.34)	0.20
3	−0.47	0.12	0.99	(0.87, 1.13)	−0.10	1.00	(0.87, 1.13)	0.00
4	−0.18	0.11	1.05	(0.87, 1.13)	0.80	1.03	(0.90, 1.10)	0.50
5	−1.76	0.18	0.94	(0.87, 1.13)	−0.90	0.99	(0.71, 1.29)	0.00
6	−3.39	0.37	0.91	(0.87, 1.13)	−1.40	0.98	(0.29, 1.71)	0.10
7	−0.62	0.12	1.00	(0.87, 1.13)	0.00	0.98	(0.86, 1.14)	−0.20
8	−2.41	0.23	1.04	(0.87, 1.13)	0.70	0.98	(0.58, 1.42)	0.00
9	0.33	0.10	0.96	(0.87, 1.13)	−0.60	0.97	(0.93, 1.07)	−0.90
10	−1.90	0.19	0.89	(0.87, 1.13)	−1.80	0.95	(0.68, 1.32)	−0.30
11	−0.97	0.14	0.98	(0.87, 1.13)	−0.30	0.99	(0.82, 1.18)	−0.10
12	−0.97	0.14	0.82	(0.87, 1.13)	−2.90	0.93	(0.82, 1.18)	−0.80
13	0.77	0.10	0.96	(0.87, 1.13)	−0.50	0.96	(0.94, 1.06)	−1.40
14	−0.59	0.12	0.98	(0.87, 1.13)	−0.30	0.99	(0.86, 1.14)	−0.20
15	−0.29	0.11	0.84	(0.87, 1.13)	−2.50	0.91	(0.89, 1.11)	−1.60
16	1.31	0.10	0.95	(0.87, 1.13)	−0.70	0.96	(0.94, 1.06)	−1.40
17	1.27	0.10	0.98	(0.87, 1.13)	−0.30	0.99	(0.94, 1.06)	−0.50
18	1.14	0.10	0.98	(0.87, 1.13)	−0.30	0.98	(0.95, 1.05)	−0.60
19	1.15	0.10	1.01	(0.87, 1.13)	0.20	1.01	(0.95, 1.05)	0.30
20	1.14	0.10	1.02	(0.87, 1.13)	0.30	1.02	(0.95, 1.05)	0.60
21	2.45	0.12	0.96	(0.87, 1.13)	−0.50	0.99	(0.87, 1.13)	−0.20
22	2.19	0.11	1.14	(0.87, 1.13)	2.10	1.07	(0.89, 1.11)	1.30
23	1.62	0.10	1.20	(0.87, 1.13)	2.90	1.14	(0.93, 1.07)	3.70
24	2.20 *	0.10	1.12	(0.87, 1.13)	1.80	1.06	(0.89, 1.11)	1.00

Note. *n*_1_ = 470. *b* = item difficulty, SE = standard error; MNSQ = mean square; CI = confidence interval; ZSTD = z-standardised fit. * parameter estimate constrained.

**Table 3:** Item parameters and fit indices for all items of the second version of the matrix reasoning test.

Item	*b*	SE	Unweighted Fit			Weighted Fit		
			MNSQ	CI	ZSTD	MNSQ	CI	ZSTD
2	−2.39	0.20	1.06	(0.87, 1.13)	0.80	1.01	(0.67, 1.33)	0.10
3	−1.42	0.14	1.01	(0.87, 1.13)	0.10	1.00	(0.82, 1.18)	0.00
4	−0.49	0.12	0.97	(0.86, 1.14)	−0.30	0.98	(0.91, 1.09)	−0.50
5	−0.92	0.13	1.03	(0.85, 1.15)	0.50	1.02	(0.87, 1.13)	0.30
6	−3.07	0.28	0.85	(0.86, 1.14)	−2.20	0.94	(0.50, 1.50)	−0.20
7	−0.63	0.12	0.97	(0.85, 1.15)	−0.40	0.98	(0.89, 1.11)	−0.30
8	−2.70	0.24	0.99	(0.85, 1.15)	−0.10	0.96	(0.59, 1.41)	−0.10
9	−0.97	0.13	1.00	(0.85, 1.15)	0.00	0.99	(0.86, 1.14)	−0.10
10	−2.21	0.19	0.91	(0.86, 1.14)	−1.20	0.93	(0.69, 1.31)	−0.40
11	−1.68	0.16	0.85	(0.86, 1.14)	−2.10	0.94	(0.78, 1.22)	−0.50
12	−1.26	0.14	0.82	(0.86, 1.14)	−2.60	0.92	(0.83, 1.17)	−0.90
13	0.28	0.11	0.96	(0.86, 1.14)	−0.50	0.97	(0.95, 1.05)	−1.20
14	−1.11	0.13	1.03	(0.86, 1.14)	0.50	1.02	(0.85, 1.15)	0.20
15	−1.56	0.15	0.91	(0.87, 1.13)	−1.30	0.97	(0.80, 1.20)	−0.30
16	0.79	0.11	0.96	(0.86, 1.14)	−0.50	0.97	(0.94, 1.06)	−0.90
17	0.87	0.11	0.94	(0.86, 1.14)	−0.80	0.96	(0.94, 1.06)	−1.30
18	0.24	0.11	0.97	(0.86, 1.14)	−0.40	0.97	(0.95, 1.05)	−1.30
19	0.15	0.11	1.03	(0.86, 1.14)	0.50	1.03	(0.94, 1.06)	1.00
20	0.42	0.11	0.98	(0.85, 1.15)	−0.20	0.99	(0.95, 1.05)	−0.50
21	2.09	0.14	0.91	(0.86, 1.14)	−1.20	0.97	(0.82, 1.18)	−0.20
22	1.35	0.12	1.09	(0.86, 1.14)	1.20	1.06	(0.90, 1.10)	1.10
23	1.25	0.12	1.13	(0.85, 1.15)	1.70	1.08	(0.91, 1.09)	1.70
24	1.18	0.11	1.03	(0.86, 1.14)	0.40	1.01	(0.92, 1.08)	0.30
25	0.97	0.11	1.01	(0.85, 1.15)	0.10	1.01	(0.93, 1.07)	0.30
26	0.47	0.11	1.00	(0.86, 1.14)	0.00	1.00	(0.95, 1.05)	−0.20
27	−1.49	0.15	0.98	(0.86, 1.14)	−0.20	1.00	(0.80, 1.20)	0.10
28	0.07	0.11	0.98	(0.85, 1.15)	−0.30	0.98	(0.94, 1.06)	−0.80
29	−0.15	0.11	0.98	(0.86, 1.14)	−0.30	0.97	(0.93, 1.07)	−0.90
30	0.84	0.11	1.02	(0.86, 1.14)	0.30	1.02	(0.94, 1.06)	0.60
31	0.43	0.11	0.99	(0.85, 1.15)	−0.10	0.99	(0.95, 1.05)	−0.40
32	−1.67	0.16	0.88	(0.86, 1.14)	−1.60	0.96	(0.78, 1.22)	−0.30
33	2.17	0.15	1.15	(0.85, 1.15)	1.90	1.03	(0.81, 1.19)	0.30
34	1.45	0.12	1.01	(0.85, 1.15)	0.20	0.99	(0.89, 1.11)	−0.10
35	0.82	0.11	0.95	(0.85, 1.15)	−0.60	0.95	(0.93, 1.07)	−1.60
36	−1.30	0.14	0.86	(0.85, 1.15)	−2.00	0.93	(0.82, 1.18)	−0.80
37	−1.92	0.17	0.88	(0.86, 1.14)	−1.60	0.97	(0.74, 1.26)	−0.20
38	1.51	0.12	1.18	(0.86, 1.14)	2.30	1.09	(0.89, 1.11)	1.50
39	−0.40	0.11	0.97	(0.86, 1.14)	−0.40	0.98	(0.91, 1.09)	−0.50
40	−0.01	0.11	1.04	(0.86, 1.14)	0.50	1.03	(0.94, 1.06)	1.00
41	0.26	0.11	0.97	(0.86, 1.14)	−0.30	0.98	(0.95, 1.05)	−0.80
42	1.60	0.12	1.08	(0.86, 1.14)	1.20	1.04	(0.88, 1.12)	0.70
43	−1.77	0.17	0.83	(0.85, 1.15)	−2.40	0.95	(0.76, 1.24)	−0.40
44	1.56	0.12	1.13	(0.85, 1.15)	1.70	1.08	(0.88, 1.12)	1.30
45	−1.43	0.15	0.87	(0.86, 1.14)	−1.80	0.94	(0.81, 1.19)	−0.60
46	1.69	0.13	1.07	(0.86, 1.14)	1.00	1.04	(0.87, 1.13)	0.60
47	−0.63	0.12	1.02	(0.86, 1.14)	0.20	1.00	(0.89, 1.11)	0.00
48	1.30	0.12	1.02	(0.86, 1.14)	0.30	1.02	(0.90, 1.10)	0.40
49	2.58	0.17	1.67	(0.86, 1.14)	7.70	1.11	(0.76, 1.24)	0.90
50	1.57	0.12	1.08	(0.86, 1.14)	1.10	1.06	(0.88, 1.12)	0.90
51	0.81	0.11	0.96	(0.86, 1.14)	−0.50	0.97	(0.94, 1.06)	−1.10
52	1.01	0.11	0.99	(0.86, 1.14)	−0.10	0.98	(0.93, 1.07)	−0.40
53	−1.07	0.13	1.03	(0.86, 1.14)	0.40	1.00	(0.85, 1.15)	0.00
54	−1.26	0.14	0.86	(0.86, 1.14)	−2.00	0.92	(0.83, 1.17)	−0.90
55	1.09	0.11	1.07	(0.86, 1.14)	0.90	1.06	(0.92, 1.08)	1.50
56	0.99	0.11	0.99	(0.86, 1.14)	−0.10	0.99	(0.93, 1.07)	−0.20
57	1.44	0.12	1.03	(0.86, 1.14)	0.50	1.02	(0.89, 1.11)	0.40
58	0.04	0.11	0.93	(0.86, 1.14)	−0.90	0.94	(0.94, 1.06)	−1.90
59	0.10	0.11	1.03	(0.86, 1.14)	0.40	1.03	(0.94, 1.06)	1.00
60	0.12 *	0.11	1.07	(0.85, 1.15)	0.90	1.05	(0.94, 1.06)	1.80

Note. *n*_2_ = 425. *b* = item difficulty, SE = standard error; MNSQ = mean square; CI = confidence interval; ZSTD = z-standardised fit. * parameter estimate constrained.

**Table 4:** Mantel-Haenszel DIF results for cohort, gender, and grade Level.

Item	Cohort		Gender		Grade Level	
	Δ_MH_	*p*_adj_	Δ_MH_	*p*_adj_	Δ_MH_	*p*_adj_
2	0.88	0.93	0.57	0.98	1.81	0.99
3	3.17	0.24	−1.00	0.93	1.35	0.99
4	−0.83	0.33	0.13	0.98	0.16	0.99
5	−1.02	0.33	−0.08	0.98	−0.80	0.99
6	1.32	0.98	−1.12	0.98	−0.03	0.99
7	0.82	0.93	0.36	0.98	0.38	0.99
8	0.91	0.94	−0.31	0.98	0.18	0.99
9	−1.15	0.24	0.60	0.83	−0.90	0.99
10	−1.00	0.55	0.56	0.98	−0.25	0.99
11	0.97	0.99	0.14	0.93	0.08	0.99
12	−1.61	0.08	0.48	0.93	−0.56	0.99
13	0.44	0.98	0.28	0.98	0.13	0.99
14	−1.38	0.24	0.05	0.98	−0.53	0.99
15	−2.56	0.08	1.43	0.74	−0.75	0.99
16	−0.89	0.53	0.13	0.98	−0.10	0.99
17	−1.03	0.53	1.06	0.74	−0.19	0.99
18	−1.21	0.24	0.12	0.98	−0.73	0.99
19	−0.25	0.79	−0.23	0.98	−0.37	0.99
20	−1.05	0.47	0.27	0.98	−0.91	0.99
21	−0.90	0.61	0.42	0.98	−0.04	0.99
22	−0.43	0.72	−0.02	0.99	0.09	0.99
23	−0.11	0.98	−0.25	0.98	0.48	0.99
24	−0.13	0.98	0.84	0.83	0.48	0.99
25	−0.89	0.52	0.16	0.98	0.22	0.99
26	−0.77	0.53	0.44	0.98	−0.21	0.99
27	2.11	0.72	0.00	0.98	0.99	0.99
28	−0.76	0.53	0.81	0.74	−0.13	0.99
29	−0.91	0.47	0.73	0.93	−0.02	0.99
30	−0.85	0.47	0.45	0.93	−0.28	0.99
31	0.55	0.78	0.58	0.74	0.56	0.99
32	−0.03	0.73	−0.59	0.98	−0.28	0.99
33	0.63	0.91	0.75	0.93	0.32	0.99
34	−0.51	0.78	0.65	0.74	0.00	0.99
35	−0.69	0.55	−0.28	0.99	0.40	0.99
36	−0.09	0.94	−0.17	0.98	−0.12	0.99
37	0.14	0.94	−0.75	0.99	−0.04	0.99
38	−0.90	0.53	0.01	0.99	−0.04	0.99
39	−0.10	0.73	0.80	0.83	0.51	0.99
40	−1.01	0.37	0.60	0.93	−1.27	0.99
41	−0.54	0.68	0.14	0.98	0.21	0.99
42	−0.54	0.72	−0.26	0.98	0.78	0.99
43	0.44	0.94	0.27	0.98	−0.02	0.99
44	−0.27	0.91	0.63	0.74	−0.44	0.99
45	−0.52	0.73	−0.38	0.98	−0.46	0.99
46	0.21	0.98	0.20	0.98	0.33	0.99
47	0.31	0.94	−0.34	0.98	0.22	0.99
48	−0.34	0.73	0.10	0.98	0.40	0.99
49	−0.79	0.56	0.35	0.98	0.45	0.99
50	0.21	0.99	−0.04	0.99	0.62	0.99
51	−0.22	0.77	−0.38	0.98	0.20	0.99
52	−0.73	0.53	0.39	0.93	0.85	0.99
53	−0.29	0.73	0.26	0.98	0.14	0.99
54	−1.15	0.37	−0.78	0.98	−0.54	0.99
55	−1.47	0.24	−0.19	0.98	−0.23	0.99
56	−0.25	0.78	−0.33	0.98	0.31	0.99
57	0.71	0.78	0.29	0.98	0.35	0.99
58	−0.26	0.73	0.85	0.74	0.40	0.99
59	−0.54	0.56	0.11	0.98	0.43	0.99
60	−0.15	0.98	−0.11	0.98	0.46	0.99

Note. Cohort: focal = control group students, reference = enrichment students; Gender: focal = female, reference = male; Grade level: focal = secondary grade level, reference = primary grade level. Δ_MH_ = ETS delta transform of the Mantel-Haenszel odds ratio; positive values indicate the item favours the focal group, negative values favour the reference group. *p*_adj_ = Benjamini-Hochberg adjusted *p*-values from the Mantel-Haenzsels chi-square test of uniform DIF (adjustment applied within each grouping variable).

**Table 5:** Correlations between *θ*_MR_, *θ*_SI_, and IQ disaggregated by grade level.

Variable	1	2	3
1. *θ*_MR_	–	.33 ***	.43 ***
2. *θ*_SI_	.59 ***	–	.27 ***
3. IQ	.55 ***	.44 ***	–

Note. *θ*_MR_ = person’s ability level in the matrix reasoning test; *θ*_SI_ = person’s ability level in the scientific inquiry assessment. The results for the primary grade level (*n* = 212) are shown above the diagonal. The results for the secondary grade level (*n* = 128) are shown below the diagonal. *** *p* < .001.

**Table 6:** Latent regression Rasch model for matrix reasoning ability by cohort.

Regression Variable	Estimate (Logits)	SE	z	*p*	95% CI
Intercept	0.509	0.20	16.42	<.001	[0.448, 0.570]
Cohort	−0.332	0.12	−4.74	<.001	[−0.469, −0.195]

Note. Group coding: 1 = control students, 0 = enrichment students (reference). Negative coefficients for the group variable indicate lower mean ability in the control group.

**Table 7:** Multi-Group Rasch Model of Matrix Reasoning by Cohorts.

Enrichment		Control		*d*
M	SD	M	SD	
0.00	0.54	−0.31	0.61	−0.53

Note. *d* = standardised mean difference on the theta scale. Results were linked using the Stocking-Lord method. Means (and SDs) are reported in logits on the enrichment-group metric (enrichment = reference).

## Data Availability

The original data presented in the study are openly available in Mendeley Data at https://data.mendeley.com/datasets/cx7zwx7k66/1 (accessed on 12 April 2026). The developed test instrument is available from the corresponding author upon reasonable request for research purposes. Upon completion of the dissertation, the full instrument, including the manual, IRT parameters, and scoring code, will be made publicly available via the Open Science Framework (OSF).

## Abbreviations

The following abbreviations are used in this manuscript:

*g*	General Intelligence
*G_f_*	Fluid Intelligence
PNA	Psychometric Network Analysis
STEM	Science, Technology, Engineering, and Mathematics
CPS	Complex Problem Solving
IQ	Intelligence Quotient
DBR	Design-Based Research
VerE model	Modell zur Vernetzung der Erkenntnisgewinnung in Biologie und Chemie [Model of Cross-Linking Scientific Inquiry between Biology and Chemistry]
KR-20	Kruder-Richardson Formula
IRT	Item Response Theory
EAP	Expected a Posteriori
WLE	Mean Weighted Likelihood Estimation
MNSQ	Mean-Square
ZSTD	z-standardised statistics
PCA	Principal component analysis
DIF	Differential Item Functioning
MH	Mantel-Haenszel
*θ*_MR_	Person Ability Parameter in Matrix Reasoning Tasks
*θ*_SI_	Person Ability Parameter in Scientific Inquiry
SEM	Standard Error of the Mean
